# COVID-19 Message: Necessity of Attention to Passive Defense in Emerging Diseases

**DOI:** 10.30476/ijcbnm.2021.88819.1549

**Published:** 2021-04

**Authors:** Vahid Zamanzadeh, Majid Purabdollah, Mostafa Ghasempour

**Affiliations:** 1 Department of Medical Surgical Nursing, School of Nursing and Midwifery, Tabriz University of Medical Sciences, Tabriz, Iran

**DEAR EDITOR**

Due to the unique genetic structure of coronaviruses, as well as their ability to reproduce and easily spread, the emergence of the novel coronavirus disease (COVID-19) has not been unexpected for virologists. Based on the information collected to-date on COVID-19, due to the complex structure of the virus, high risk of inter-human transmission, presence of asymptomatic or mildly-symptomatic carriers, and progression of the condition to respiratory distress and 5-10% mortality, the occurrence of this pandemic can be defined as a kind of perfect storm. ^1^

The recent Covid-19 pandemic, in addition to its negative economic, social, political, sports and religious implications, has posed many challenges for the healthcare sector: increase in the need for medical staff, increase in the cost of personal prevention and protection equipment, cost of diagnosing and treating patients, need for more Intensive Care Unit beds and ventilated beds, and increase in the mortality rate in the community. Even the continuation of this process could impose irreparable costs on governments. The World Health Organization (WHO) has also expressed concern about the continuing prevalence of Covid-19 disease and warned about the burden of the disease. Influenza, with an annual mortality rate of 0.023%, kills 31,9213 individuals out of 1.39 billion people a year. It is estimated that Covid-19 disease with a mortality rate of 3.7% will cause 52 million deaths (approximately two-thirds of the number of victims of World War II) worldwide. ^2^ As for the challenges of international peace and security, the Security Council has identified “contagious diseases” as one of the current challenges. ^3^ The WHO considers it necessary to ensure the highest level of health, justice in the health sector, reduction of the risk of health-related factors, and especially the development of the necessary infrastructure to eradicate infectious diseases. ^2^

Therefore, it is not surprising that the issue of health is on the agenda of the Security Council, and assuming that any interpretation of the source of the spread of infectious diseases, whether natural or bioterrorism, can certainly be considered a threat to global security. ^3^ Governments are committed to enforcing human rights, which requires funding, providing facilities, and reforming the infrastructures, and creating conditions, so that the individuals can enjoy their rights as much as possible. ^3^

Therefore, it seems that in the third millennium we must prepare ourselves for biological defense. Empowering the country is probably the best way to deal with such crises. In medical sciences, prevention has always been a priority. It is obvious that the capital of a country is its human resources, and the protection of the health of the body and soul of the people of that society is the most important strategy of any country. ^4^ In our country (Iran), in addition to military organizations, non- military organizations are also responsible for managing bioterrorism crisis in the form of passive defense; meanwhile, the health care system plays an important role in managing such crises. ^5^ Passive defense is a set of unarmed measures that, regardless of the source of the threat, lead to a reduction in the vulnerability of manpower, facilities, and equipment and promote national security. ^5^ In our country, the problems of passive defense include the lack of research centers, indicators to determine the optimal state of passive defense in the field of health, a comprehensive operational plan, managerial independence and funds and sufficient budget; and poor structure. ^4 , 5^

It seems that in addition to natural disasters, there is a war between the genetic material of the virus and the human intellect, and in order to win this battle, in addition to knowing enough about this invasive and contagious virus, it is necessary to make logical decisions with a preventive approach. Iran is the tenth country in the world and the fourth largest country in the Middle East in terms of natural disasters. ^6^ Therefore, considering the general policies of the system based on passive defense of the country, special attention should be paid to the role and position of passive defense in managing the country’s crisis.

It is logical that modern man should try to find a way to deal with disasters using new technologies and save his country and people from death and destruction, or at least reduce the damage. In this regard, by increasing the budget and strengthening the system, specialized promotion of executive managers’ knowledge in the field of passive defense management, education and awareness of the people, and especially the readiness of health and treatment system in terms of prevention, diagnosis and treatment equipment, vaccinate the population to some extent.
